# Cardiovascular magnetic resonance TE-averaged susceptibility weighted imaging of reperfused intramyocardial hemorrhage

**DOI:** 10.1186/1532-429X-17-S1-Q37

**Published:** 2015-02-03

**Authors:** James W Goldfarb, Usama Hasan

**Affiliations:** 1 St Francis Hospital, Roslyn, NY, USA

## Background

In the setting of acute myocardial infarction (AMI), therapeutic and spontaneous reperfusion of ischemic myocardium can lead to interstitial intramyocardial hemorrhage (IMH) which is associated with microvascular obstruction (MVO) and subsequent adverse clinical outcomes. Imaging without contrast agents (native imaging) can be used in AMI patients for additional myocardial tissue characterization. Native T1 and T2 weighted imaging and quantitative measurements have been reported to detect myocardial edema and depict the myocardial area at risk. IMH affects T1, T2 and T2* relaxation as well as susceptibility and the feasibility of several MR image contrasts (T1, T2, T2* and gradient-echo phase) has been demonstrated for the depiction of IMH. Susceptibility weighted imaging (SWI) uses a type of image contrast different from traditional spin density, T1 or T2 weighted MR imaging.

In the present work, we report our experience with myocardial SWI imaging (combined gradient-echo magnitude and phase imaging) for the detection of IMH. We propose TE image averaging and gray-scale inversion as a means of providing a single image with good image SNR and excellent contrast for the detection of IMH.

## Methods

Eleven AMI subjects were studied at 1.5T before contrast agent administration with a dark blood double inversion recovery multiple spoiled gradient-echo sequence (12 echo times, 2.4 - 15.5 ms;1.2 ms spacing) and conventional CINE and late gadolinium-enhanced (LGE) imaging. Magnitude, susceptibility weighted and TE-averaged images were reconstructed from raw data k-space data. Contrast and signal-difference-to-noise were measured and compared between SWI methods for IMH detection. Volumes of LGE, IMH and MVO were measured and reported as a percentage of LV myocardial volume.

## Results

**Figure 1:**
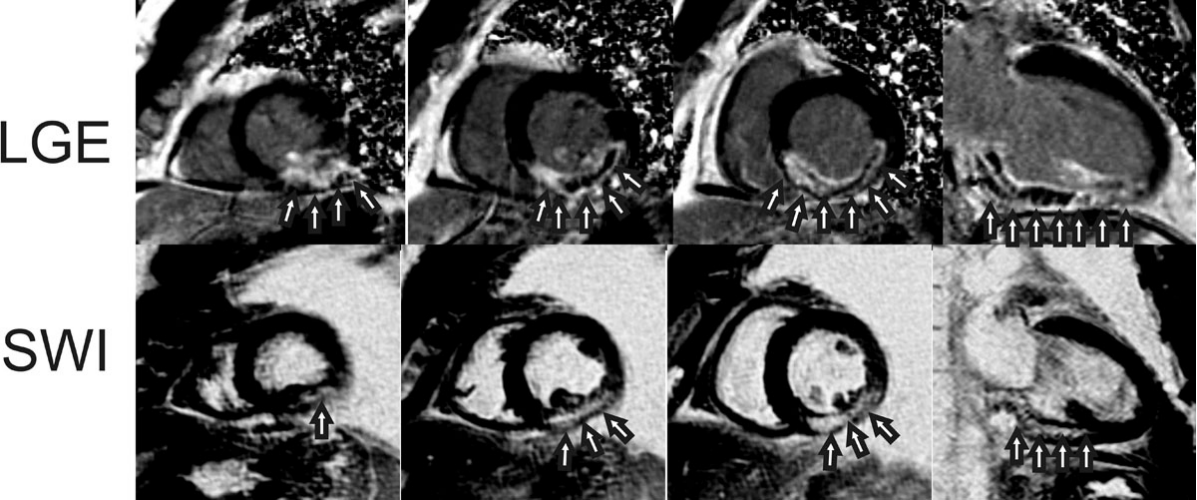
Short-axis and two chamber images from a patient with an acute inferior lateral wall myocardial infarction. LGE images show circumscribed transmural infarction with MVO (Top row, arrows). Precontrast SWI images show a hyperintense lesion consistent with IMH in the same area as MVO defined by LGE imaging (bottom row, arrows).

**Figure 2:**
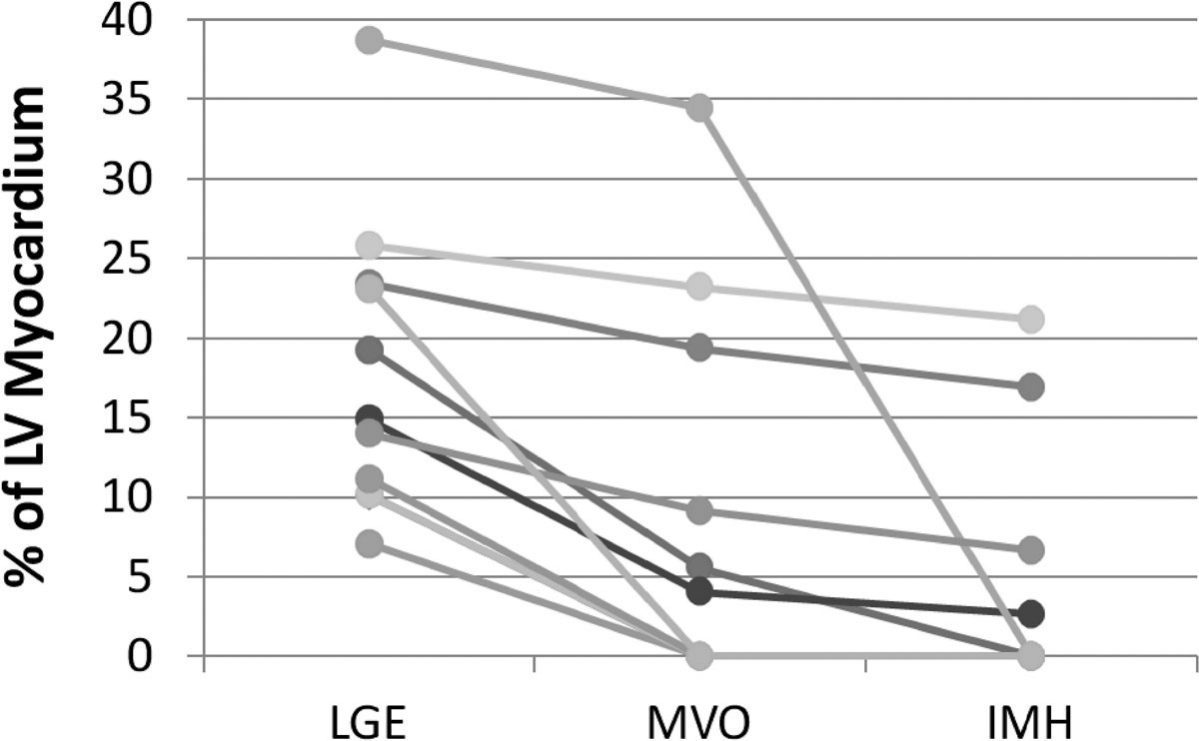
Comparison among LGE, MVO and IMH volumes. Graph shows the left ventricular percentage of late gadolinium enhancement (LGE), microvascular obstruction (MVO) and intramyocardial hemorrhage (IMH) for acute myocardial infarction study participants.

There were six patients with microvascular obstruction (MVO) and four patients with IMH detected by TE-averaged SWI imaging. TE-averaged SWI images from a representative AMI subject with IMH are given in Figure 1. All patients with IMH on SWI scans had MVO on late gadolinium-enhanced (LGE) imaging. There was a three-fold increase in IMH contrast with SWI compared to magnitude images. IMH contrast decreased and signal-to-noise increased with increased TE averages. Results of IMH, LGE and MVO volume measurements are displayed in Figure 2. The trend was that larger infarcts had MVO and IMH, but this was not true in all cases as the largest infarct had no evidence of IMH.

## Conclusions

TE-averaged SWI imaging is a promising method for myocardial tissue characterization in the setting of AMI for the detection of IMH. Along with gray-scale colormap inversion, it combines not only magnitude and phase information, but also images across TEs to provide a single image sensitive to IMH with characteristics similar to LGE imaging.

## Funding

American Heart Association scientist development grant (0635029N).

